# Ignorance and Disease

**Published:** 1871-06

**Authors:** 


### IGNORANCE AND DISEASE

Are closely associated in all ages, places, and nations; hence whatever efforts are made for promoting a higher intelligence among the masses, surely tend to a higher hygiene: and those men who are leaders in this work, merit public and national gratitude. In order to promote a more general education among the masses in the city of New York, the State authorities have appropriated nearly three millions of dollars to be spent annually for school-houses and books and teachers for the children of the city, and it is now within the power of every New York family to give every child, boy and girl, a superior education, without a single dollar's expense. We know a young lady, now abroad, who never went to any other than the Twelfth Street public school of New York city, who speaks and reads and writes the German language with more than general native accuracy; she is well versed in German literature, is a good Italian scholar, a fine singer, and an admirable performer on the piano; and all within three years after leaving school. Among our citizens who have been longest and foremost in the organization of public schools, are Gerard, Roosevelt, Vanvorst, Bernard, Wood, Sands, Smythe, and others. In a recent address, indicating research, ability, and power, by Judge Vanvorst, one of the commissioners of public instruction, at a meeting for the organization of the Board, the statement was made, on authority, from official documents, that of the 38,000,000 of people in France, fifteen million could neither read nor write; no wonder such a people are incapable of self-government; no wonder that brutality and blood, despotism, public plunder and individual license, run riot in beautiful Paris, and a whole nation is engaged in drinking its own blood. And as population increases in this country, and competition in trade and labor becomes more severe, a greater moral power, a greater mental might, from the education of the masses, becomes an increasing necessity; and Judge Vanvorst but enunciated a great truth in his admirable address when he says, "In States where the whole power rests with the people, and where both the form of the government, and the method of the execution of its authority, and the designation of the agents who are to enforce its execution, rest upon their choice and will, it is of the first importance that they be well informed, and intellectually and morally educated, as well as physically developed."

